# Trends in the epidemiological and microbiological profiles of infectious keratitis in southeastern Brazil

**DOI:** 10.5935/0004-2749.20230050

**Published:** 2023

**Authors:** Carolina Saliba de Freitas, Marcelo Oliveira Mesquita, Mayara Seyko Kaczorowski Sasaki, Alice Zaidan Azevedo, Artur Willian Caldeira Abreu Veloso, Marco Antônio Guarino Tanure, Daniel Vítor Vasconcelos-Santos

**Affiliations:** 1 Departamento de Oftalmologia e Otorrinolaringologia e Laboratório de Ciências Visuais, Faculdade de Medicina, Universidade Federal de Minas Gerais, Belo Horizonte, MG, Brazil.; 2 Hospital São Geraldo, Hospital das Clínicas, Universidade Federal de Minas Gerais, Belo Horizonte, MG, Brazil.; 3 Programa de Pós-graduação em Ciências da Saúde, Infectologia e Medicina Tropical, Universidade Federal de Minas Gerais, Belo Horizonte, MG, Brazil.

**Keywords:** Keratitis, Eye infections, bacterial, Anti-bacterial agents, Ceratite, Infecções oculares bacterianas, Antibacterianos

## Abstract

**Purpose:**

To investigate the antibiotic susceptibility as well as the clinical, epidemiological, and microbiological profiles of microbial keratitis.

**Methods:**

This was a longitudinal retrospective study, and we retrospectively reviewed medical and laboratory records from 2015 to 2019.

**Results:**

In total, 380 pathogens (321 bacteria and 59 fungi) were isolated from the corneas of 352 patients. *Staphylococcus* species (45%) were most abundant within the organisms that were isolated, followed by *Pseudomonas* (18.4%), fungi (15.5%), *Streptococcus* (7.9%), and *Serratia* species (3.2%). The isolated gram-positive bacteria were not resistant to amikacin or vancomycin, although 14.8% of the gram-positive isolates were resistant to ciprofloxacin (p<0.05). All the gram-negative isolates were susceptible to amikacin. Male patients represented 62.8% of the 129 cases with accessible clinical data. The mean age of the patients was 53.17 ± 21 years. The time to presentation (from onset of symptoms) was 14.9 ± 19.4 days (median: 7 days). Large ulcers (>5 mm in any dimension) were present in 49.6% (64 eyes) of the cases. The duration of treatment was 49 ± 45.9 days (median: 38 days). Direct ocular trauma was reported by 48 (37.2%) patients, and 15 patients (11.6%) reported using contact lenses. For 72 (55.8%) patients, topical treatment had been previously prescribed, and 16 (12.4%) patients reported using other classes of drugs. Hospitalizations were required for 79 (61.2%) patients, and in terms of major complications, 53 (41.1%) patients had corneal perforations. A total of 40 patients (31%) underwent tectonic penetrating keratoplasty, and 28 (21.7%) developed secondary glaucoma. A progression to endophthalmitis occurred in 8 (6.2%) patients, with 50% of those patients’ (3.1% of the total) endophthalmitis evolving to evisceration. The patients’ microbial keratitis was largely treated empirically, with 94 (72.9%) patients prescribed moxifloxacin and 56 (43.4%) prescribed ciprofloxacin before receiving their culture results.

**Conclusions:**

For the most part, our hospital treated patients with severe microbial keratitis. Despite identifying gram-positive bacteria in most of the isolates, we also frequently identified gram-negative rods and fungi. Our susceptibility results support prescribing a combination of vancomycin and amikacin as an effective empirical therapeutic regimen to treat microbial keratitis.

## Abstract (pt)

**Objetivo:**

Investigar a susceptibilidade a antibió­ticos, o perfil clínico, epidemiológico e microbiológico das ce­ratites infecciosas.

**Métodos:**

Estudo retrospectivo longitu­dinal. Registros médicos e laboratoriais de 2015 a 2019 fo­ram revisados retrospectivamente.

**Resultados:**

Trezentos e oitenta patógenos (321 bactérias e 59 fungos) foram isolados das córneas de 352 pacientes. As espécies de *Staphylococcus* foram os microorganismos mais isolados (45%), seguidos de *Pseudomonas* (18,4%), fungos (15,5%), *Streptococcus* (7,9%) e *Serratia* (3,2%). Não houve resistência das bactérias Gram-positivas à amicacina ou vancomicina, enquanto 14,8% isolados Gram-positivos foram resistentes à ciprofloxacina (p<0,05). Todos os organismos Gram-negativos eram suscetíveis à amicacina. Pacientes do sexo masculino representaram 62,8% de 129 casos com dados clínicos acessíveis. A média de idade foi 53,17 ± 21 anos. O tempo até a apresentação (desde o início dos sintomas) foi de 14,9 ± 19,4 dias (mediana: 7 dias). Úlceras grandes (>5mm em qualquer extensão) representaram 49,6% (64 olhos) dos casos. A duração do tratamento foi de 49 ± 45,9 dias (mediana: 38 dias). Trauma ocular direto foi relatado por 48 (37,2%) pacientes e uso de lentes de contato por 15 (11,6%) pacientes. Foi prescrito tratamento prévio para 72 (55.8%) pacientes. Outras classes de medicamentos foram prescritas para 16 (12.4%). Setenta e nove (61,2%) pacientes tiveram que ser hospitalizados. Como complicações maiores, 53 (41,1%) pacientes apresentaram perfuração corneana, 40 pacientes (31%) foram submetidos à ceratoplastia penetrante tectônica e 28 (21,7%) desenvolveram glaucoma secundário. Oito pacientes (6,2%) evoluíram para endoftalmite. O tratamento empírico da ceratite microbiana foi amplamente empregado, com 94 (72,9%) pacientes em uso de moxifloxacina e 56 (43,4%) em uso de ciprofloxacina antes do resultado da cultura.

**Conclusões:**

Nosso hospital tratou predominantemente de pacientes com úlceras microbianas graves. Embora bactérias Gram-po­sitivas constituíssem a maioria dos isolados, bacilos e fungos Gram-negativos também foram frequentemente identificados nas ceratites microbianas. Os resultados de suscetibilidade sugerem a combinação de vancomicina e amicacina como um regime terapêutico empírico eficaz para essa condição grave com risco de perda visual permanente.

## INTRODUCTION

Microbial keratitis is an acute and potentially sight-threatening ocular condition caused by bacteria, fungi, protozoa, and viruses. Pathogenic microbes often cause ulcerative necrotizing inflammation in the corneal stroma, which may progress to corneal perforation^(1)^. The annual incidence of microbial keratitis is estimated to be higher in developing countries (789:100,000)^(2)^ than in developed countries (11:100,000)^(3)^, and studies have shown that it has been increasing over the years^(4)^. Microbial keratitis rarely affects intact eyes as the cornea has a natural resistance to infection with the corneal epithelium acting as the main barrier to foreign pathogens;^(5)^ however, predisposing factors, including trauma, wearing contact lenses, previous corneal surgery, and the prolonged use of corticosteroids, may weaken the defense mechanisms of the ocular surface, allowing invasion of the cornea by microorganisms^(6,7)^.

The etiology of microbial keratitis varies depending on geographic, demographic, behavioral, and economic factors^(8)^. Because of the acute nature of microbial keratitis and frequent delays in securing microbiological results, the initial treatment of microbial keratitis is often empiric, consisting of an intensive regimen of topical antibiotics. Before antibiotic treatment begins, however, corneal scrapes are generally recommended to orient further treatment by identifying the pathogen and its antibiotic susceptibility^(9)^.

The main purpose of this study was to investigate the antibiotic susceptibility of the pathogens responsible for microbial keratitis as well as the clinical, epidemiological, and microbiological profiles of patients with microbial keratitis at Hospital São Geraldo/HC-UFMG, a referral center in southeastern Brazil. By identifying the trends in the incidence and susceptibility of microbial keratitis, we may help clinicians determine effective treatment and clinical approaches for patients with infectious keratitis.

## METHODS

This was an institutional review board (IRB)-approved retrospective study conducted at the Cornea and Ocular External Diseases Department of Hospital São Geraldo, the eye hospital affiliated with Universidade Federal de Minas Gerais. The study protocol followed the tenets of the Declaration of Helsinki.

A search of the computerized corneal ulcer database initially identified clinically suspected cases of infectious keratitis that had been microbiologically investigated at the referral center between January 2015 and May 2019. These cases had been diagnosed as “corneal ulcers,” based on observations of epithelial defects overlying stromal infiltrates made during patients’ slit-lamp exams. We reviewed the cases of microbial keratitis to determine the isolated organisms and their respective antibiotic susceptibility profiles.

Based on previous reports, we collected data on the age, sex, ulcer dimensions (large ulcers were defined as ˃5 mm in any extent)^(10)^, risk factors (history of trauma, use of contact lenses, previous ocular surface disease), clinical conditions (diabetes, hypertension) and previous topical treatments of the patients with culture-positive cases. Furthermore, we also looked into certain outcome variables of the patients such as major complications (perforation or glaucoma or the need for tectonic keratoplasty, endophthalmitis, evisceration, hospitalization, and/or treatment with oral analgesics). Treatment duration, defined from the beginning of treatment until the absence of stromal infiltration, was measured in days. Data on treatment with antibiotic drops were also collected/analyzed. Initial and final visual acuity was measured using the Snellen scale.

Patients with negative cultures and those who had other suspicious clinical diagnoses (herpes, *Acanthamoeba* sp.) were excluded as validated protocols to analyze these microorganisms were not available in our laboratory.

Following their clinical examinations, patients were subjected to microbiological investigations. Corneal scrapings were performed on all the patients with presumed microbial keratitis. The scrapings were obtained from the ulcer’s edges and base using a spatula blade, and one drop of proxymetacaine 5 mg/ml was used for topical anesthesia. The scraped corneal tissue was placed on glass slides for Gram and Giemsa staining. The tissues were also inoculated in solid (blood, chocolate, and *Sabouraud* agar plates/tubes) and liquid media (thioglycolate broth) and subsequently sent to our microbio­logy laboratory for analysis according to predefined protocols. The bacterial and fungal isolates were identified, and their antibiotic susceptibility profiles were determined using standard microbiological procedures. Smear and culture results were recorded along with the assessed clinical details in our corneal ulcer database.

Descriptive statistics were calculated, and all statistical analyses were performed using R software (R Development Core Team 2019) with nonparametric tests. P<0.05 was regarded as statistically significant.

## RESULTS

In the study, 545 patients with presumed microbial keratitis underwent corneal scrapings, and pathogens were recovered in 352 of these patients (64.6%). Bacterial keratitis accounted for 293 of the positive growths (77.1% of all isolates), with a total of 28 patients (7.4%) having multiples isolates from the same sample (only bacteria). The 59 remaining positive cultures were fungi (15.5% of all isolates).

The total number of gram-positive and gram-negative isolates was 206 (54.2%) and 115 (30.3%), respectively. The frequency of all the isolated microorganisms is shown in table 1.

**Table 1:** Microorganisms isolated from corneal ulcers

Isolate	n	% (95% CI)
Gram-positive bacteria	206	54.2 (49.1-59.3)
*Staphylococcus sp.*	171	45.0 (39.9-50.2)
*Streptococcus sp.*	30	7.9 (5.4-11.1)
*Enterococcus sp.*	5	1.3 (0.4-3.0)
Gram-negative bacteria	115	30.3 (25.7-35.1)
*Pseudomonas sp.*	70	18.4 (14.7-22.7)
*Serratia sp.*	12	3.2 (1.6-5.5)
*Escherichia sp.*	7	1.8 (0.7-3.8)
*Citrobacter sp.*	5	1.3 (0.4-3.0)
*Proteus sp.*	5	1.3 (0.4-3.0)
*Klebsiella sp.*	4	1.1 (0.3-2.7)
*Morganella sp.*	4	1.1 (0.3-2.7)
*Enterobacter sp.*	3	0.8 (0.2-2.3)
*Acinetobacter sp.*	1	0.3 (0.0-1.5)
*Burkholderia sp.*	1	0.3 (0.0-1.5)
*Haemophilus sp.*	1	0.3 (0.0-1.5)
*Strenotrophomonas sp.*	1	0.3 (0.0-1.5)
Filamentous fungi	55	14.5 (11.1-18.4)
*Fusarium sp.*	40	10.5 (7.6-14.1)
*Aspergillus sp.*	11	2.9 (1.5-5.1)
*Paecilomyces sp.*	3	0.8 (0.2-2.3)
*Scedosporium sp.*	1	0.3 (0.0-1.5)
Yeast-like fungi	4	1.0 (0.3-2.7)
*Sporothrix sp.*	2	0.5 (0.1-1.9)
*Dematiaceous fungi*	1	0.3 (0.0-1.5)
*Candida sp.*	1	0.3 (0.0-1.5)

95% CI: 95% confidence interval

*Staphylococcus* sp. was the most prevalent cultured bacteria (45% of all growths), and hence, it was the most common gram-positive bacteria, accounting for 83% of all the gram-positive isolates. The most common gram-negative bacterial species was *Pseudomonas aeruginosa* (18.4% of all growths), accounting for 60.9% of all the gram-negative isolates.

Fungi accounted for 15.5% of all the positive-growth cultures. Filamentous fungi were the most prevalent, with *Fusarium* sp. being responsible for 67.8% of all the fungal growths and 10.5% of the total growths. Only four cultures identified yeasts. In all, 24 patients had trauma with vegetal matter, and 50% of these patients had positive cultures for *Fusarium* sp.

### Antibiotic susceptibility of gram-positive microorganisms

The susceptibility of the gram-positive microorganisms to vancomycin was 100% (Table 2), and the resistance of these gram-positive microorganisms to ciprofloxacin was 14.8%, with *Staphylococcus* sp. showing the higher rates of resistance. The gram-positive microorganisms’ resistance to other antibiotics was variable (Table 3).

**Table 2:** Antibiotic resistance patterns of gram-positive and gram-negative bacteria isolated from corneal ulcers

	Antibiotic	No. of resistant isolates	Total isolates	% (95% CI)
Gram-Positive	Amikacin	0	32	0.0 (0.0-10.9)
	Ampicillin	1	5	20.0 (0.5-71.6)
	Chloramphenicol	0	28	0.0 (0.0-12.3)
	Ciprofloxacin	25	169	14.8 (9.8-21.1)
	Gentamicin 120	2	6	33.3 (4.3-77.7)
	Gentamicin	11	162	6.8 (3.4-11.8)
	Tobramycin	0	3	0.0 (0.0-70.8)
	Vancomycin	0	46	0.0 (0.0-7.7)
Gram-Negative	Amikacin	0	108	0.0 (0.0-3.4)
	Ampicillin	25	34	73.5 (55.6-87.1)
	Ciprofloxacin	2	106	1.9 (0.2-6.6)
	Gentamicin	4	107	3.7 (1.0-9.3)
	Tobramycin	0	7	0.0 (0.0-41.0)

**Table 3:** Antibiotic resistance patterns of microorganisms isolated from corneal ulcers

	Antibiotic	Resistant isolates (n)	Total isolates (n)	% (CI)
*Staphylococcus sp.*	Amikacin	0	30	0.0 (0.0-11.6)
	Ciprofloxacin	27	169	15.0 (9.9-21.3)
	Gentamicin	21	170	6.9 (3.5-12.0)
	Vancomycin	0	17	0.0 (0.0-19.5)
*Streptococcus sp.*	Chloramphenicol	0	27	0.0 (0.0-12.8)
	Vancomycin	0	25	0.0 (0.0-13.7)
*Citrobacter sp.*	Amikacin	0	5	0.0 (0.0-52.2)
	Ampicillin	5	5	100.0 (39.0-100)
	Ciprofloxacin	0	5	0.0 (0.0-52.2)
	Gentamicin	0	5	0.0 (0.0-52.2)
*Escherichia sp.*	Amikacin	0	7	0.0 (0.0-41.0)
	Ampicillin	1	7	14.3 (0.4-57.9)
	Ciprofloxacin	0	7	0.0 (0.0-41.0)
	Gentamicin	0	6	0.0 (0.0-45.9)
*Enterobacter sp.*	Amikacin	0	3	0.0 (0.0-70.8)
	Ampicillin	3	3	100.0 (29.2-100.0)
	Ciprofloxacin	0	3	0.0 (0.0-70.8)
	Gentamicin	0	3	0.0 (0.0-70.8)
*Proteus sp.*	Amikacin	0	5	0.0 (0.0-52.2)
	Ampicillin	1	4	25.0 (0.6-80.6)
	Ciprofloxacin	0	5	0.0 (0.0-52.2)
	Gentamicin	0	5	0.0 (0.2-52.2)
*Pseudomonas sp.*	Amikacin	0	69	0.0 (0.2-52.2)
	Ciprofloxacin	1	66	1.5 (0.0-8.2)
	Gentamicin	4	68	4.5 (0.9-12.5)
	Tobramycin	0	7	0.0 (0.0-41.0)
*Serratia sp.*	Amikacin	0	11	0.0 (0.0-28.5)
	Ampicillin	7	7	100.0 (59.0-100.0)
	Ciprofloxacin	0	10	0.0 (0.0-30.8)
	Gentamicin	1	12	8.3 (0.2-38.5)
*Klebsiella sp.*	Amikacin	0	4	0.0 (0.0-60.2)
	Ampicillin	4	4	100.0 (39.8-100.0)
	Ciprofloxacin	0	4	0.0 (0.0-60.2)
	Gentamicin	0	4	0.0 (0.0-60.2)
*Morganella sp.*	Amikacin	0	4	0.0 (0.0-60.2)
	Ampicillin	4	4	100.0 (39.8-100.0)
	Ciprofloxacin	1	4	25.0 (0.6-80.6)
	Gentamicin	1	4	0.0 (0.0-70.8)
*Enterococcus sp.*	Ampicillin	1	5	20.0 (0.5-71.6)
	Gentamicin	2	5	40.0 (5.3-85.3)
	Vancomycin	0	4	0.0 (0.0-60.2)

### Antibiotic susceptibility of gram-negative microorganisms

The susceptibility of the gram-negative isolates to ciprofloxacin and amikacin was 98.1% and 100%, res­pectively. (Table 2). When analyzing the microorganisms’ specific resistance patterns, we found that 100% of the *Serratia marcescens* and *Klebsiella* sp. isolates were susceptible to ciprofloxacin. The gram-negative microor­ganisms’ resistance to other antibiotics was variable (Table 3).

### Demographics, risk factors, and outcome measures

Of the 352 culture-positive patients, complete clinical information was accessible for 129 patients (36.6%). Male patients represented 62.8% of the cases. The patients’ mean age was 53.17 ± 21 years. The right eye was affected in 49.6% (64 eyes) and the left eye was affected in 50.4% (65 eyes) of the patients. The time to presentation (from the onset of symptoms) ranged from 14.9 ± 19.4 (median: 7) days. Large ulcers (>5 mm in any dimension) represented 49.6% (64 eyes) of the cases. Previous topical treatment had been prescribed for 72 (55.9%) patients prior to their referral to our corneal service (Table 4). Potential risk factors and comorbidities are described in Table 4. The best-corrected VA at presentation ranged from no light perception (NLP) to 20/20, with 66 (51.2%) patients presenting with VA ≤ hand movements. The patients’ microbial keratitis was frequently treated empirically while waiting for culture results, with 94 (72.9%) patients were prescribed moxifloxacin and 56 (41.3%) were prescribed ciprofloxacin. Because of the severity of the cases, a significant proportion of the patients experienced complications (Table 4). The duration of treatment was 49 ± 45.9 days (median: 38 days). The best-corrected VA at the patients’ last follow-up visits (49 ± 45.9 days) ranged from NLP to 20/20, with 95 (73.6%) patients still keeping VA ≤ hand movements and only 20 (15.5%) patients evolving a VA equal to or better than 20/63.

**Table 4:** Clinical data of patients with culture-proven infectious keratitis presenting to a corneal referral service in southeastern Brazil

		n	%
Risk factors	Trauma	24	18.6
	Trauma with vegetable matter	24	18.6
	Use of contact lens	15	11.6
	Ocular surface disease	1	0.8
Previous topical treatment	None	57	44.1
	Antibiotics	38	29.5
	Antibiotics + steroids	13	10.1
	Only steroids	5	3.9
	Other classes	16	12.4
Comorbidities	Hypertension	35	27.1
	Diabetes	11	8.5
Complications	Need for hospitalization	57	44.1
	Need to use oral analgesics	38	29.5
	Corneal Perforation	53	41.1
	Tectonic keratoplasty	40	31
	Secondary glaucoma	28	21.7
	Endophthalmitis	8	6.2
	Evisceration	4	3.1

## DISCUSSION

This is the largest report on the spectrum of organisms involved in microbial keratitis and their antibiotic susceptibilities in Minas Gerais, the second most populous state in Brazil. Besides identifying the microorganisms isolated from the study’s patients, we also characterized the clinical features and outcomes of a subset of these patients. As the incidence, distribution, and resistance patterns of isolates from microbial keratitis may vary and change over time, microbiological surveys are thus valuable tools to provide information to clinicians, helping them to more effectively treat and manage this vision-threatening acute condition.

In our study, male patients represented 62.8% of the cases, probably because of male’s greater exposure to risk factors, particularly to ocular trauma, which was reported in more than one third of the patients (as was observed in another Brazilian study^(11)^). Patients are referred to our hospital from all parts of the state of Minas Gerais and even from surrounding states. Patients having to travel long distances to reach our hospital’s corneal center and experiencing delays with referrals may have led to patients not receiving immediate care. We identified 72 patients that were being treated by clinicians elsewhere or had self-medicated before they were referred to our center; of these 72 patients, 33 of them had received questionable prescriptions (steroids, vasoconstrictors, and antibiotics in subtherapeutic doses). Because of possible delay in referral to our hospital, the study’s patients may have arrived in an advanced stage of keratitis. Furthermore, their conditions may have been exacerbated by the questionable prescriptions given to them during their initial treatments. Thus, the severity and size of the patient’s ulcers and their necessity for longer treatments, hospitalizations, and penetrating keratoplasty for the management of high rates of perforation may be explained. Both the patients’ initial and final VAs were indicative of the severe visual impairment they experienced and demonstrated how devastating infectious keratitis can be.

Ocular trauma (either accidental or surgical), the wearing of contact lenses, the prolonged use of corticosteroids, and systemic comorbidities have been identified as risk factors of infectious keratitis in many other studies^(10,12)^ and were also identified in our research. Ocular surface disease was not remarkable in our study, possibly because the patients in our study were unaware of its presence or likelihood (Many patients had never been to an ophthalmologist before).

We confirmed microbial infections by culture in 64.6% of the cases, with 35.6% of the smears being gram-negative, which is consistent with the literature’s data^(13)^. In al., 39.6% of patients reported prior use of topical antibiotics (with or without steroids), which may also have contributed to the resulting negative cultures. We did not have data regarding the previous treatments of patients with negative-culture results.

*Staphylococcus* sp. was the most frequently cultured organism. This finding is similar to other Brazilian studies that found the prevalence of *Staphylococcus* sp. to be 41%-51.7% of the gram-positive cocci in their corneal samples^(13,14)^.

*Pseudomonas aeruginosa* was the second most prevalent microorganism isolated from corneal ulcers in our study (18.4%), corroborating the results of other studies in Brazil and in the United States^(10,15)^. This gram-negative rod is regarded in some studies as the main bacteria implicated in corneal infections^(10-12)^. A fifteen-year study from St. Louis, MO documented a progressive rise in the incidence of *Pseudomonas* sp. over the years, which the study’s authors attributed to an increase in contact lens-related keratitis^(10)^. In our study, only 11.6% of the patients reported wearing contact lenses, possibly because the hospital’s patients come from poor areas, and contact lenses are still relatively expensive for a significant part of Brazil’s population. *Pseudomonas* sp. is associated particularly with more severe keratitis. In their study, however, Ibrahim et al. did not find *Pseudomonas* sp. as a leading cause of keratitis at another referral center in Brazil. Their findings were justified based on the few patients wearing contact lenses in their sample^(14)^. On the other hand, another Brazilian study from a private practice center showed a higher frequency of *Pseudomonas* sp. (29%), attributing its high frequency to the large proportion of the study’s patients wearing contact lenses^(11)^.

We observed a high, albeit not surprising incidence of fungal keratitis at our center, particularly infections involving filamentous fungi. The proportion of fungi we observed (15.5%) in our cases of microbial keratitis was comparable with what other referral centers in Brazil^(14)^ and other developing countries had observed^(9)^. In Dallas, TX a study found 14%-15% of microbial keratitis cases involved fungi, which is similar to the 16% found in the St. Louis, MO study^(10,16)^. This incidence of fungal keratitis can be even higher (46%-82%), as shown in studies conducted in China and in other developing countries with larger rural populations^(17)^.

It is known that epidemiology of mycotic keratitis also varies depending on climate conditions. In temperate climates (i.e., central Europe, England, and the northern United States), *Candida* sp. is the most frequently isolated fungi, while in tropical countries/areas (i.e., South America, southern Florida, Japan, and South Africa), filamentous fungi predominate, particularly *Fusarium* sp. Filamentous fungi are mainly related to trauma with vegetal matter^(17,18)^, which is what we observed in our study as 12 of the 24 patients who suffered from this kind of trauma had positive cultures for *Fusarium* sp.

Microorganism’s emerging resistance to antimicrobial drugs is indeed a global problem, but this problem has regional nuances, varying upon local microbial spectrum, their antibiotic susceptibility, and other variables including practice patterns, and even self-medication. Periodic susceptibility testing should be performed to ensure that the antimicrobials being used are still providing effective coverage against isolates of bacterial keratitis, especially with clinicians often empirically prescribing antibiotics before receiving culture results^(17)^.

By analyzing antibiotic susceptibility profiles of gram-positive and gram-negative organisms isolated from corneal ulcers and the most frequently prescribed antibiotics for corneal conditions, we were able to make a few observations regarding bacteria’s antibiotic resistance. First, gram-positive bacteria were not resistant to chloramphenicol. This broad-spectrum antibiotic fell into disuse in the field of ophthalmology beginning in the mid-1980s because of ocular pathogens’ increasing resistance to it. It has been hypothesized that because chloramphenicol was in disuse for so many years, bacteria’s susceptibility to it may have improved^(17)^. However, resistance rates of ocular pathogens to chloram­phenicol are still relatively high in some countries, reaching 47.9% in the United Kingdom for instance^(19)^.

Second, the percentage of gram-positive bacteria that were resistant to ciprofloxacin was 14.8%. Since this fluoroquinolone’s initial use, there have been numerous reports of bacteria’s increasing resistance to ciprofloxacin, especially in cases of methicillin-resistant *S. aureus* keratitis^(20)^. Studies in the United States have reported that approximately 80% of ocular isolates of methicillin-resistant *S. aureus* were resistant to this fluo­roquinolone^(21)^. In 2004, the in vitro susceptibility of *S. aureus* and *S. pneumoniae* to ciprofloxacin recovered from ocular samples at a referral center in São Paulo was 92.6% and 90.9%, respectively^(17)^, which is comparable to the 93.7% susceptibility described in the UK^(19)^. We found that only 1.9% of the gram-negative isolates were resistant to ciprofloxacin, which is consistent with the expected spectrum of coverage of this quinolone. Of note, all the *Klebsiella* sp. and *Serratia* sp. isolates were susceptible to ciprofloxacin. We observed that only 1.5% of *Pseudomonas* sp. was resistant to ciprofloxacin, but recent literature has suggested that *Pseudomonas* sp. has decreasing susceptibility to ciprofloxacin, with 92.96%-96.1% of *Pseudomonas* sp. showing resistance^(17,22)^. Presently, fourth-generation quinolones, like moxifloxacin and gatifloxacin, are becoming the first choice for the empiric treatment of microbial keratitis. Their extended spectrum of activity encompasses gram-positive species (including *staphylococci, streptococci,* and *enterococci*) and anaerobes, which is far superior to ciprofloxacin’s spectrum of activity. However, although widely used, fourth-generation fluoroquinolones are not FDA approved for the treatment of bacterial keratitis^(7)^. Some studies have shown an increase in antibiotic resistance even to fourth-generation quinolones^(8,23)^, with some of these studies describing a dramatic increase in the proportion of moxifloxacin-resistant organisms isolated over a three-year period^(24)^. Unfortunately, testing for the susceptibility to these antibiotics was not possible at our laboratory.

We also did not isolate any gram-positive species that were resistant to vancomycin. This result is similar to what was observed in another study in the United States^(10)^ and a little different than a 16-year Toronto study that found only 0.4% of gram-positive species were resistant to vancomycin. These data suggest that vancomycin may be an effective antibiotic choice to treat severe gram-positive keratitis.

Gram-negative species showed a good susceptibility profile to gentamicin, which is in line with other studies^(17,25,26)^. We believe gentamicin’s low rates of susceptibility can be explained by its toxicity, and thus infrequent use, but it could be used as an option in the event of extensive antibiotic resistance.

An important finding from this study was that all gram-negative species were sensitive to amikacin, as was seen in another study in Spain^(25)^. A 12-year analysis in the United Kingdom found that 97.9% of gram-negative species were susceptible to this antibiotic^(26)^.

Our study had several limitations, including its retrospective design and small representation of some microbial species. In addition, the small representation of some clinical variables precluded a statistical analysis of risk factors, for instance. By concentrating on culture isolates, we left out *Acanthamoeba* sp., a rare but important etiology of severe microbial keratitis observed in referral centers worldwide. Moreover, only major, frequently prescribed antibiotics in ophthalmology were studied and even some of them, particularly fourth-ge­neration quinolones, could not be included in the susceptibility testing at our reference lab. As an important academic center in the state/country, our university-ba­sed eye hospital has a referral bias toward more complicated/severe/refractory cases. Nevertheless, our center still can be compared with other similar centers in Brazil and worldwide. Finally, antibiotic susceptibility based on in vitro testing may differ from clinical results because penetration of the antibiotic and host factors also influence clinical outcomes.

In conclusion, our sample mostly comprised cases of severe infectious keratitis, with long-term complex management and frequent complications resulting in visual impairment despite treatment. Bacteria represented the main etiology of infectious keratitis in our center, with *Staphylococcus* sp. being the most frequently isolated microorganism. The combined use of fortified vancomycin and amikacin provided effective treatment for 100% of the gram-positive and gram-negative isolates respectively, and no observable resistance to these antibiotics was seen in this study.

## References

[1] Wong RL, Gangwani R, Yu LW, Lai JS (2012). New treatments for bacterial keratitis. J Ophthalmol.

[2] Upadhyay MP, Karmacharya PC, Koirala S, Shah DN, Shakya S, Shrestha JK (2001). The Bhaktapur eye study: ocular trauma and antibiotic prophylaxis
for the prevention of corneal ulceration in Nepal. Br J Ophthalmol.

[3] Erie JC, Nevitt MP, Hodge DO, Ballard DJ (1993). Incidence of ulcerative keratitis in a defined population from
1950 through 1988. Arch Ophthalmol.

[4] Gopinathan U, Sharma S, Garg P, Rao GN (2009). Review of epidemiological features, microbiological diagnosis and
treatment outcome of microbial keratitis: experience of over a
decade. Indian J Ophthalmol.

[5] Yildiz EH, Airiani S, Hammersmith KM, Rapuano CJ, Laibson PR, Virdi AS (2012). Trends in contact lens-related corneal ulcers at a tertiary
referral center. Cornea.

[6] Whitcher JP, Srinivasan M (1997). Corneal ulceration in the developing world-a silent
epidemic. Br J Ophthalmol.

[7] Lin A, Rhee MK, Akpek EK, Amescua G, Farid M, Garcia-Ferrer FJ, American Academy of Ophthalmology Preferred Practice Pattern Cornea
and External Disease Panel (2019). Bacterial keratitis preferred practice patterni. Ophthalmology.

[8] Peng MY, Cevallos V, McLeod SD, Lietman TM, Rose-Nussbaumer J (2018). Bacterial keratitis: isolated organisms and antibiotic resistance
patterns in San Francisco. Cornea.

[9] Paro G, Zanardo S, Chicani CF, Gomes JA, Lima Filho AA, Cunha MC (1998). Correlação entre bacterioscopia e cultura nas
úlceras de córnea e as implicações do uso de
antibiótico prévio. Rev Bras Oftalmol.

[10] Hsu HY, Ernst B, Schmidt EJ, Parihar R, Horwood C, Edelstein SL (2019). Laboratory results, epidemiologic features, and outcome analyses
of microbial keratitis: a 15-year review from St. Louis. Am J Ophthalmol.

[11] Farias R, Pinho L, Santos R (2017). Epidemiological profile of infectious keratitis. Rev Bras Oftalmol.

[12] Duarte MC, Becker GN, Muller GG, Tuon FF (2020). Infectious keratitis in southern Brazil: a comparison culture
negative and culture positive patients. Rev Bras Oftalmol.

[13] Marujo FI, Hirai FE, Yu MC, Hofling-Lima AL, Freitas D, Sato EH (2013). Distribuição das ceratites infecciosas em hospital
terciário no Brasil. Arq Bras Oftalmol.

[14] Ibrahim MM, Vanini R, Ibrahim FM, Martins WP, Carvalho RT, Castro RS (2011). Epidemiology and medical prediction of microbial keratitis in
southeast Brazil. Arq Bras Oftalmol.

[15] Wakisaka E, Ferreira MA, Rocha FJ, Freitas LL, Guidugli T, Lima AL, Lima ALHd (1990). Cultura de material provindo de úlceras de córnea em
laboratório de referência. Arq Bras Oftalmol.

[16] Truong DT, Bui MT, Cavanagh HD (2018). Epidemiology and outcome of microbial keratitis: private
university versus urban public hospital care. Eye Contact Lens.

[17] Chalita MR, Höfling-Lima AL, Paranhos A Jr, Schor P, Belfort R Jr (2004). Shifting trends in in vitro antibiotic susceptibilities for
common ocular isolates during a period of 15 years. Am J Ophthalmol.

[18] Tanure MA, Cohen EJ, Sudesh S, Rapuano CJ, Laibson PR (2000). Spectrum of fungal keratitis at Wills eye hospital, Philadelphia,
Pennsylvania. Cornea.

[19] Shalchi Z, Gurbaxani A, Baker M, Nash J (2011). Antibiotic resistance in microbial keratitis: ten-year experience
of corneal scrapes in the United Kingdom. Ophthalmology.

[20] Leibowitz HM (1991). Clinical evaluation of ciprofloxacin 0.3% ophthalmic solution for
treatment of bacterial keratitis. Am J Ophthalmol.

[21] Asbell PA, Sanfilippo CM, Pillar CM, DeCory HH, Sahm DF, Morris TW (2015). Antibiotic Resistance Among Ocular Pathogens in the United
States: Five-Year Results From the Antibiotic Resistance Monitoring in
Ocular Microorganisms (ARMOR) Surveillance Study. JAMA Ophthalmol.

[22] Lichtinger A, Yeung SN, Kim P, Amiran MD, Iovieno A, Elbaz U (2012). Shifting trends in bacterial keratitis in Toronto: an 11-year
review. Ophthalmology.

[23] Jhanji V, Sharma N, Satpathy G, Titiyal J (2007). Fourth-generation fluoroquinolone-resistant bacterial
keratitis. J Cataract Refract Surg.

[24] Oldenburg CE, Lalitha P, Srinivasan M, Rajaraman R, Ravindran M, Mascarenhas J (2013). Emerging moxifloxacin resistance in Pseudomonas aeruginosa
keratitis isolates in South India. Ophthalmic Epidemiol.

[25] Chen A, Prajna L, Srinivasan M, Mahalakshmi R, Whitcher JP, McLeod S (2008). Does in vitro susceptibility predict clinical outcome in
bacterial keratitis?. Am J Ophthalmol.

[26] Tan SZ, Walkden A, Au L, Fullwood C, Hamilton A, Qamruddin A (2017). Twelve-year analysis of microbial keratitis trends at a UK
tertiary hospital. Eye (Lond).

